# VisionaryVR: An Optical Simulation Tool for Evaluating and Optimizing Vision Correction Solutions in Virtual Reality

**DOI:** 10.3390/s24082458

**Published:** 2024-04-11

**Authors:** Benedikt W. Hosp, Martin Dechant, Yannick Sauer, Björn Severitt, Rajat Agarwala, Siegfried Wahl

**Affiliations:** 1 ZEISS Vision Science Lab, Institute for Ophthalmic Research, University of Tübingen, Maria-von-Linden Straße 6, 72076 Tübingen, Germany; 2 Interaction Centre, University College London, 66-72 Gower Street, London WC1E 6EA, UK; 3 Carl Zeiss Vision International GmbH, Turnstraße 27, 73430 Aalen, Germany

**Keywords:** virtual reality, vision, correction, simulation, tool

## Abstract

In the rapidly advancing field of vision science, traditional research approaches struggle to accurately simulate and evaluate vision correction methods, leading to time-consuming evaluations with limited scope and flexibility. To overcome these challenges, we introduce ‘VisionaryVR’, a virtual reality (VR) simulation framework designed to enhance optical simulation fidelity and broaden experimental capabilities. VisionaryVR leverages a versatile VR environment to support dynamic vision tasks and integrates comprehensive eye-tracking functionality. Its experiment manager’s scene-loading feature fosters a scalable and flexible research platform. Preliminary validation through an empirical study has demonstrated VisionaryVR’s effectiveness in replicating a wide range of visual impairments and providing a robust platform for evaluating vision correction solutions. Key findings indicate a significant improvement in evaluating vision correction methods and user experience, underscoring VisionaryVR’s potential to transform vision science research by bridging the gap between theoretical concepts and their practical applications. This validation underscores VisionaryVR’s contribution to overcoming traditional methodological limitations and establishing a foundational framework for research innovation in vision science.

## 1. Introduction

Vision impairments represent a significant global health concern, impacting millions of individuals. Refractive errors, encompassing conditions like myopia (nearsightedness), hyperopia (farsightedness), astigmatism, and presbyopia, impair the eye’s ability to focus light directly onto the retina, leading to blurred vision. Current estimates regarding the trend of myopia predict 3.4 billion people, or half the world’s population, will have myopia by 2050 [1]. Beyond refractive errors, cataracts pose a substantial challenge, clouding the eye’s lens and progressively impairing vision. Age-related macular degeneration (AMD) is another critical issue characterized by the loss of central vision and significantly hampering daily activities like reading and facial recognition. Its prevalence is estimated at 7.7 million people worldwide [2]. Glaucoma, encompassing a group of conditions that damage the optic nerve, often due to high intraocular pressure, poses a severe risk of vision loss and blindness if left untreated. It is estimated to affect 79.6 million people worldwide and remains the leading cause of irreversible blindness [3]. Additionally, developmental disorders like amblyopia (Lazy Eye) and strabismus (Crossed Eyes) can lead to lifelong vision impairment if not addressed in their early stages. Collectively, these conditions represent the most prevalent vision impairments known to medical science. Understanding the etiology of these impairments and discovering effective correction, treatment, or healing methods is imperative. However, transitioning from conceptual research to publicly available solutions is time-intensive.

In Paul Hibbard’s book [4], the connection between VR and vision science and how we can use VR to improve vision science research is explained in detail. Hibbard shows the importance and strength of combining them. Specifically for vision science, no foundational tool helps scientists build their experiments. While there are approaches to using VR for vision science, they fail to support new experiments and most often have a specific purpose. BlueVR is specifically implemented to raise awareness for color blindness [5], Albrecht et al. [6] built a headset to augment peripheral vision exclusively called MoPedT. Their display toolkit can be built with off-the-shelf products, allowing designers and researchers to develop new peripheral vision interaction and visualization techniques. While MoPedT has a high external validity as it augments reality, it might help in real-life situations. However, developing new optical methods heavily relies on high experimental control to understand the dynamics under controlled circumstances, which cannot be achieved in real-life augmentations. Another closer related paper from Barbieri et al. [7] describes Realter, a reality-altering simulator for raising awareness of low-vision diseases like age-related macular degeneration, glaucoma, and hemianopsia. Their main focus is on simulating specific diseases rather than creating a foundational architecture for other researchers. Realter works as an extended reality device to simulate the diseases mentioned above while allowing the collection of eye- and head-tracking data from an HTC Vive Pro Eye with an integrated Tobii eye-tracker. Thus, it is restricted to specific devices and diseases. Like Albrecht et al. [6], Barbieri et al. [7] focused on applications in the real world rather than on high experimental control in virtual reality. As such, both devices are great tools for applied research in the field to simulate specific diseases and conditions and collect data about human behavior but are restricted to real scenes, therefore, missing the possibilities of virtual reality. Next, other works aim to provide a simulator to improve specific ophthalmic conditions or hardware. One exemplary simulator is described in the work of Niessner et al. [8]. They simulate eye accommodation in their simulation of human vision using different surfaces. They show two ways of simulating defocus. The progressively distributed ray tracing of eye lenses guides lens manufacturers during the design process. The second approach is implemented to give customers a real-time impression of spectacles in an optician store. In their system, they additionally visualize the refractive power and astigmatism that allow a quality assessment of special-purpose lenses for sports or reading. Another work on VR simulators for vision science was published by Barbero et al. [9]. Their work shows a procedure to simulate real-world scenes through spectacle lenses. They aim to anticipate the effects of image transformation by optical defects found in spectacle lenses. They focus on blur, distortion, and chromatic aberration to evaluate ophthalmic lenses in a VR simulator.

Inspired by the works mentioned earlier, we have developed an innovative simulation tool to expedite the development, robustness, and testing ease of new optical solutions for vision correction. Our approach can be seen as a foundation for works like Niessner et al. [8] to speed up such developments in a broader range. This tool combines an accurate optical method simulation and an evaluation procedure in a controlled environment, allowing for testing and refinement before implementation in physical devices. VisionaryVR, functioning as a virtual reality simulator, can accurately replicate blur effects due to optical aberrations—flaws in the image formation process of optical systems that lead to the scattering of light rays—diminishing the sharpness of the perceived image. It can also simulate conditions such as glaucoma and age-related macular degeneration (AMD), offering a realistic portrayal of these visual impairments. While VR allows a free, natural, and explorative behavior in evaluating different conditions, it supports a high experimental control to investigate several types of vision impairment and environmental interactions scientifically. The simulation of AMD or glaucoma is crucial as it can help to understand the affected people’s behavior and create appropriate treatments or therapies (as shown in [10]). VisionaryVR represents a technological innovation and a step forward in bridging the gap between theoretical research and practical applications in vision science. Its design and capabilities have been developed to offer a comprehensive platform for simulating optical phenomena and evaluating vision correction solutions. To underscore the practical applicability and efficacy of VisionaryVR, an empirical study has been conducted and published [11], validating its utility in real-world scenarios. This study demonstrates VisionaryVR’s potential impact on the field, highlighting its role in advancing optical health and vision correction technologies through rigorous scientific validation.

Our tool is anchored in an experiment controller, providing a foundational architecture for a virtual reality simulator. This simulator enables experimenters to seamlessly integrate independently built scenes applicable to various conditions in randomized or sequential order. Additionally, the tool features an integrated questionnaire scene loader for collecting direct VR responses, enhancing the tool’s capability to evaluate and understand the user experience comprehensively. VisionaryVR is a foundational simulator for eye-tracking, focus simulation, and VR questionnaire experiments under multiple conditions. Its user-friendly and expansible design makes VisionaryVR a pivotal component in developing custom simulators for various sensors and actuators. Through this innovative tool, we aim to bridge the gap between theoretical research and practical applications, paving the way for advancements in optical health and vision correction technologies. A significant application of VisionaryVR is to act as a testing environment for devices based on optoelectronic lenses under active research (for an overview, please see the current state in [11]). Considering hardware tuning mechanisms and natural interaction modes, VisionaryVR’s modular structure facilitates comparing and evaluating different methods or conditions before their time-consuming implementation in hardware prototypes.

The paper proposes a scene-based evaluation framework that recreates natural scenarios to address the limitations of current approaches and explore the potential of virtual reality simulation for the research of ophthalmic solutions. This framework aims to comprehensively assess visual performance and convenience, simulating individuals’ real-life situations. By leveraging VR technology and the simulation tool, the paper aims to create an immersive framework that closely mimics these scenarios, facilitating precise measurement and analysis of the performance and convenience of optical methods or therapies. The objective of this work is to advance vision science research. By providing a realistic and controlled environment, the tool can potentially enhance real-world applications in various domains, including presbyopia correction, depth estimation, eye-tracking, and intention prediction. It can potentially revolutionize solutions for optical vision correction, enhancing the visual experience and quality of life for individuals across different fields, such as augmented reality, virtual reality, 3D imaging, and human–computer interaction. Through its comprehensive evaluation of a broad range of applications in optical systems research, the paper emphasizes the potential of the simulation tool in advancing the field. This tool aims to empower individuals with enhanced visual capabilities and enhance their everyday experiences.

## 2. Methods

**Figure 1:**
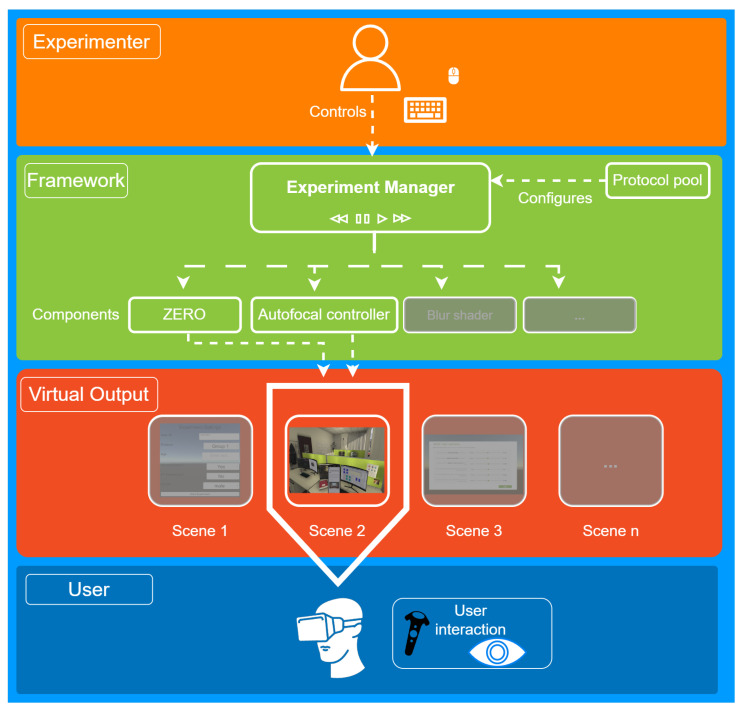
Overview of VisionaryVR.

Developing our VR simulation tool, VisionaryVR, involved a meticulous process aimed at providing a versatile and robust platform for vision science research. The VisionaryVR simulator is developed using the Unity game engine, a widely recognized and versatile platform that supports a broad range of VR devices and technologies (e.g., OpenVR, OpenXR, SteamVR). This choice of game engine ensures high performance, ease of use, and extensive support for VR development. The scripts are written in C# and accessible in the project. All used libraries are native game engine assets found in the asset store. Figure 1 shows the system divided into its key components. Several entities are involved. We start with a quick overview highlighting the key steps and their rationale behind our development process.

**Figure 2:**
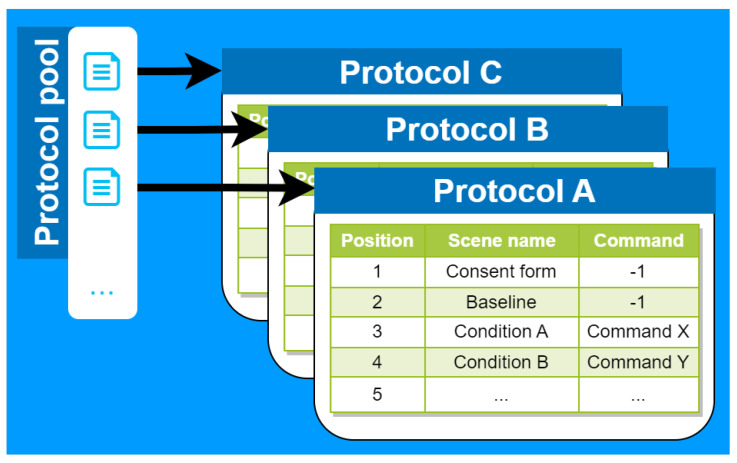
Protocol pool contains all protocols added before experimentation. Each protocol describes a sequence of parameterized scenes. This is especially useful for the randomization of conditions where each protocol represents a different sequence of conditions to reduce learning effects.

**Experiment Control:** The foundation of VisionaryVR is its experiment manager, designed to manage the flow of various simulated conditions and assessments seamlessly. This component allows researchers to orchestrate complex experimental protocols (available from the protocol pool, see Figure 2, ensuring smooth transitions between scenes and conditions. The rationale was to create a flexible environment where experiments could be customized and replicated with high fidelity, catering to diverse research needs.

**Eye-Tracking Integration:** Recognizing the crucial role of eye movement in understanding visual perception, we integrated ZERO, an open-source eye-tracking controller interface. This allows for compatibility with various VR eye-tracking devices, simplifying data collection and analysis. The choice of ZERO was motivated by its modularity and ease of use, ensuring researchers could implement eye-tracking studies without extensive technical adjustments.

**Optical Simulation:** At the core of VisionaryVR’s functionality is its ability to simulate optical aberrations and refractive errors through a depth-dependent blur technique. This approach dynamically adjusts the blur based on the object’s distance from the viewer, emulating real-life optical phenomena. The decision to use depth-dependent blur was driven by the need for a realistic representation of how different vision impairments and corrections would affect visual perception. Specifically, residual accommodation can be defined in the simulation to simulate presbyopia—decreasing the eye’s focusing capability with increasing age. The depth-dependent defocus simulation blurs objects too close for the simulated accommodative capabilities.

**Dynamic Vision Task:** We introduced a dynamic vision task to assess visual performance under varying conditions. This component challenges participants to adjust their focus between objects at different distances, simulating real-world scenarios. This feature aimed to provide a more comprehensive visual function evaluation beyond static assessments.

**Questionnaire Integration:** Understanding participants’ subjective experiences is crucial. Therefore, we incorporated a questionnaire scene loader that allows for the direct collection of VR responses. This feature facilitates gathering qualitative data on user experience and comfort, enriching the quantitative findings from the simulation.

Each component was integrated to create a holistic tool that could simulate various visual conditions, collect detailed data on participant responses, and adapt to various research requirements. The development process was iterative and continuously refined based on feedback from preliminary studies and technical testing. By doing so, we aimed to ensure that VisionaryVR would not only serve as a powerful research tool but also enhance the reproducibility and understanding of studies in vision science. The development and validation of VisionaryVR laid the groundwork for a focused empirical investigation into optimizing autofocals. Recognizing the potential of VR technology to simulate complex visual environments and corrections, this study aimed to explore different methods for tuning autofocals—a critical aspect of advancing personalized vision correction technologies. VisionaryVR, with its capacity to accurately simulate optical aberrations and dynamic vision scenarios, provided an ideal platform for this investigation. The primary objective of this study was to evaluate the efficacy of various tuning methods for autofocals, as simulated within the VisionaryVR environment. We sought to determine which tuning approaches offer the most promising results regarding visual acuity, performance, and user comfort. This study, described in our publication [11], employed a mixed-methods approach combining quantitative analysis of simulation accuracy and qualitative feedback to assess the tool’s performance and user experience. Several tuning methods for autofocals were investigated, including but not limited to manual adjustments based on user input and algorithmic adjustments informed by eye-tracking data.

We recruited 21 emmetropic participants through university networks, ensuring a diverse age sample. Participants were exposed to several vision tasks that simulated blur and correction with different tuning methods. Eye-tracking data and performance metrics were recorded in real time to quantify the effectiveness of the simulations. The study employed advanced statistical methods to analyze the data. Quantitative measures such as task completion times, accuracy rates, and eye-tracking metrics were calculated to evaluate the results. Additionally, qualitative feedback was gathered through structured interviews, focusing on user comfort. The findings from this comprehensive study underscore VisionaryVR’s significant contributions to advancing vision science research by providing a realistic and adaptable platform for simulating visual conditions and assessing optical corrections. By combining quantitative and qualitative research methods, the study validated the effectiveness of VisionaryVR and identified areas for future development, ensuring that the tool remains at the forefront of vision science research.

### 2.1. Experiment Manager

**Figure 3:**
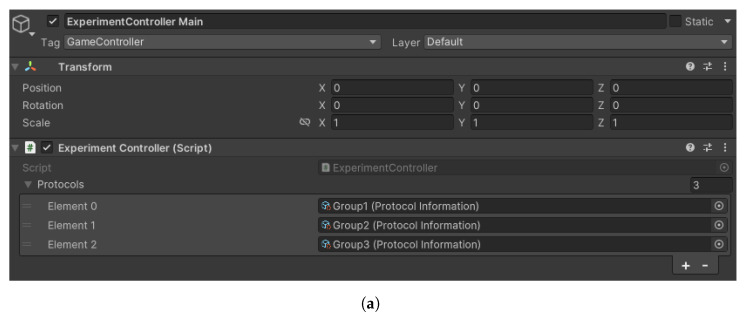

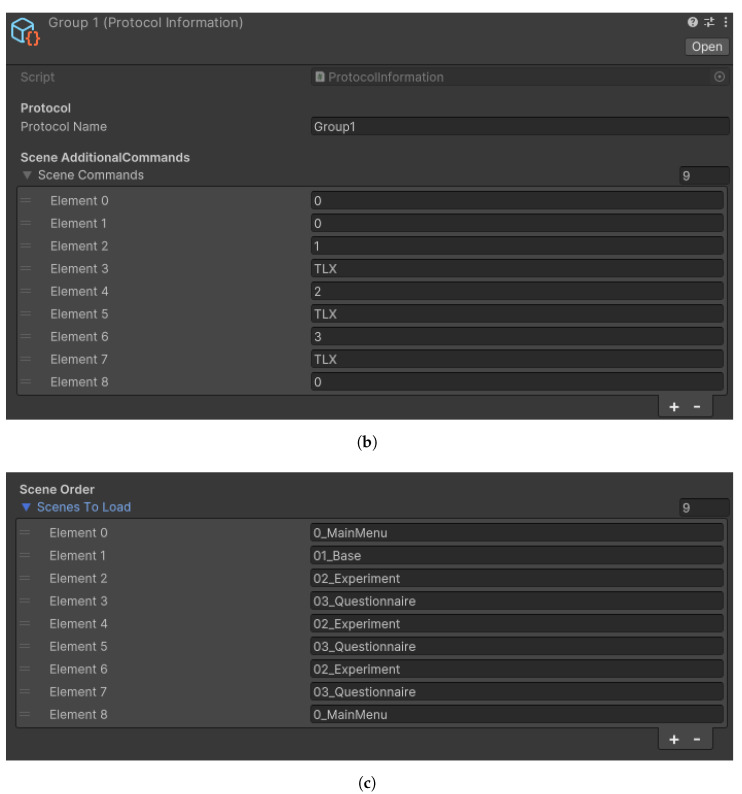
(**a**) The heart of VisionaryVR is the experiment controller that controls the main thread by loading, unloading, and parameterizing each protocol, synchronizing events and global objects across scenes. It holds the protocol pool, which can be visualized in the user interface. Each protocol object can be added to that pool in the experiment controller. The experimenter can select which protocol to run or let it decide randomly. Each protocol contains information about the parameterization and a list of scenes to load. (**b**) A parameter can be anything like a number of a string to configure a scene. Here, 0 means no additional parameter; 1, 2, and 3 parameterize the same scene 02 Experiment to load different conditions. The TLX (NASA TLX) parameter configures the 03 Questionnaire scene to load the correct questionnaire. (**c**) Next to an ordered list of scene parameters, a protocol has a corresponding list of scenes loaded in sequential order. Scenes can be loaded multiple times, e.g., when the same stimulus needs to be presented but with different conditions.

At the heart of VisionaryVR lies the experiment manager, a sophisticated scene control system for organizing and conducting experiments. Experimenters must define one or more protocols before the experiment—each represented by an object within the protocol information class. This object represents a protocol in the protocol pool as an ordered list of scenes and additional commands for scene parameterization, facilitating remotely and independently developed scenes. These are subsequently imported into Unity for integration into the experiment via the inspector view (see Figure 3). The versatility of this system is illustrated by its ability to load questionnaire scenes dynamically, with commands specifying which questionnaire to load (e.g., the NASA TLX questionnaire in scene 03, as shown in Figure 3b,c). This level of customization allows for precise experimental control and adaptability. The core functionality of the experiment controller extends beyond scene loading to include the management of game objects required throughout the experiment. It ensures the proper sequencing of scenes, offering features like repetition, restarts, and navigation between scenes, effectively managing the entire experiment through the initiation of scenes and communication with all event listeners. Both condition and questionnaire scenes are accommodated within this framework, with the ability to parameterize scenes, enhancing the tool’s flexibility. Protocols detail the sequence in which scenes are presented, allowing for the independent development and later addition of scenes to a protocol. Parameters passed for each scene facilitate a broad range of functionalities, particularly in questionnaire scenes where they determine the specific questionnaire to be loaded. An example of a protocol, “Group 1”, showcases the integration of a main menu, a baseline scene, an experimental scene, and a questionnaire scene within a single experimental run (Figure 3a). This approach underscores the tool’s ability to reutilize scene functionalities across different parameters, enabling the same scene to be employed under various conditions through distinct methodological approaches.

### 2.2. Components

The experimenter can start an experiment when the experiment manager is configured with the protocols, appropriate scenes, and parameters. Based on keyboard shortcuts, scenes can be started, stopped, repeated, and skipped. This gives the experimenter total control over the experiment procedure. Next to these foundational aspects of VisionaryVR, we included important and frequently used components that can be loaded. These components belong to the experiment manager and can be loaded in each scene individually or across scenes.

#### 2.2.1. Eye-Tracking

**Figure 4:**
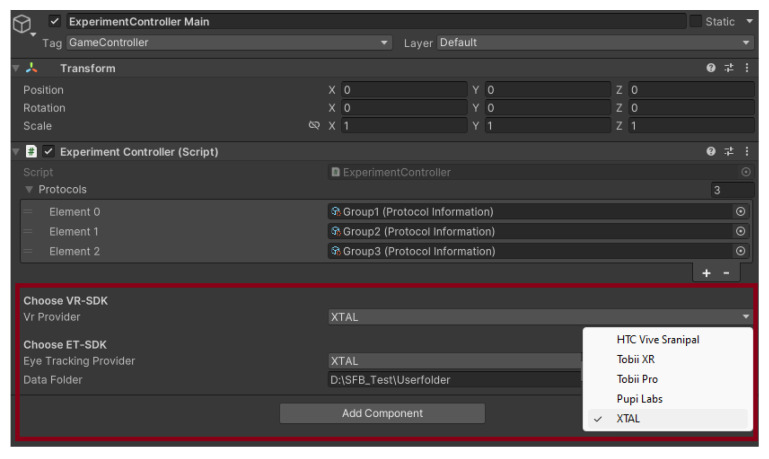
The red box shows what ZERO looks like as a component of the Experiment Controller in the inspector view of the experiment controller game object. Next to the VR Provider (VR glasses), the experimenter can decide which eye-tracking provider API to use. The currently implemented APIs are shown in a dropdown menu on the right. If the gaze files should be saved in a specific location, this can be changed in the data folder input.

In vision science, eye-tracking is an increasingly widespread tool for examinations. This technology records the direction and duration of a person’s gaze across specific areas within a visual scene, offering insights into visual focus and engagement. This method is widely employed to explore aspects of visual cognition, including attention, mental workload, and visual prominence, by analyzing pivotal eye movement patterns. The technology itself and the information on eye movements are highly investigated. To include this technology and augment the functionality of our simulation tool, we have incorporated ZERO [12], an open-source interface for eye-tracking control. ZERO furnishes a unified interface compatible with a diverse array of eye-tracking devices for virtual reality (for an example of the inspector view, see Figure 4), thereby streamlining their integration and application within VR settings. This enhancement simplifies the process of employing eye-tracking technology, making it more accessible and efficient for researchers. This integration enables seamless eye-tracking data collection and analysis, further enhancing the tool’s potential for studying human gaze behavior and interactions with optical systems. By evaluating the participants’ gaze behavior, we can assess the performance of the gaze-based interactions. ZERO can directly implement the basic functionality needed for eye-tracking studies. Next to the easy usage of the most common eye-tracking APIs, the user has a certain level of device-independent control and can quickly start and stop the eye-tracker and save the gaze files to a specified user folder location. For most studies, it can be used out of the box. If further refinement is needed, the open-source software can be adapted to one’s specific needs, as the interface is built to be highly modular and intended to be expandable by adding new eye-tracker devices via a config file. The gaze signal is saved in a generic file format that involves all the essential information needed from the eye-tracker and some unique data of specific vendors. ZERO provides calls to the interface to customize the behavior. Experimenters can start and stop the eye-tracker device and the signal sampling thread separately. If the device supports a position or eye-tracking calibration, it can be called via the ZERO interface.

#### 2.2.2. Optical Simulation

**Figure 5:**
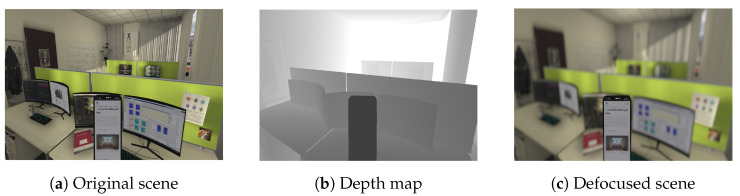
(**a**) A common office scene rendered in virtual reality. Several objects are shown in different depth planes. Ranging from close (smartphone) to far (e.g., whiteboards). (**b**) The corresponding depth map of the same scene. During rendering, the depth map can be accessed in the shader, allowing the calculation of a location-dependent blur size. For the simulation of autofocals, the depth information is also used to simulate different control algorithms that determine the target focus distance for the user’s current gaze point depending on the depth distribution in the scene (**c**) Simulated vision with an autofocal. The simulated lens is tuned to the distance of the smartphone. The rest of the scene is blurred.

Another important component of vision science is the simulation of optical aberrations. In VR, the simulation of optical aberrations utilizes a technique known as depth-dependent blur across the visual scene, as detailed in [13]. This method allows for a faithful recreation of refractive errors and various optical corrections by varying the blur level for objects at different distances from the viewer, thus mimicking the natural depth perception observed in human vision. The blur adjustment—encompassing its intensity, shape, and orientation—is dynamically calculated based on the object’s position within the scene. Such a dynamic method enables the precise simulation of lenses with spatially varying optical power. It modifies their corrective capacity across the lens surface to correct vision at different distances or angles. This is crucial for simulating progressive lenses, engineered to provide a seamless transition when viewing objects at varying distances, from near to far, by providing different optical power in different areas of the lens. An important focus of the simulation is on autofocals, based on simulating temporally varying optical power. Autofocals are eyewear designed to automatically adjust the focus for the wearer, potentially mimicking the eye’s natural ability to change focus between objects at different distances [14]. An autofocus controller for the simulated lens power is accessible via a script attached to a game object. Considering pupil size and the variable optical power of the simulated focus-tunable lens, the simulation calculates location-dependent blur size [15]. By manipulating the simulated power of the autofocal lens using arbitrary control algorithms, the simulation determines the focus distance, resulting in a natural depth of field effect where objects near the current focus distance appear sharp. This simulation empowers researchers to assess the impact of autofocal defocus on visual performance and comfort under various control mechanisms. Moreover, the simulation tool considers other relevant factors that impact focus performance, such as pupil size, lighting conditions, and environmental factors. The principle of the exemplary blur simulation is based on shaders—software to determine the final appearance of objects and scenes by manipulating lighting, color, and textures in the game engine—and, therefore, easily applicable to other effects. By incorporating these factors into the simulation, researchers can evaluate how different optical systems and control algorithms perform under varying conditions, providing valuable insights for optimizing their performance in highly natural, controlled environments. One scenario is the simulation of autofocals. To simulate autofocals, our VR headset incorporated a virtual tunable optical power, representing the optical power of a simulated autofocal lens. The range of optical power and the delay or rate of change of power of the simulated lens can be set to recreate the properties of real focus tunable lenses. The range of optical power and the delay or rate of change of power of the simulated lens can be set to recreate the properties of real focus tunable lenses [16,17]. The blur level in the field of view depended on the distance, the optical power of the tunable lens, and the participant’s pupil size. To achieve this, we calculated each pixel’s blur size (circle of confusion) based on the depth buffer. The rendered image was then blurred using a disk kernel with an extent that varied based on location. An example scene, the depth map, and the resulting view are shown in Figure 5a–c. This approach ensured that only objects near the distance fitting the current optical power appeared sharp.

### 2.3. Examples Scenes

**Figure 6:**
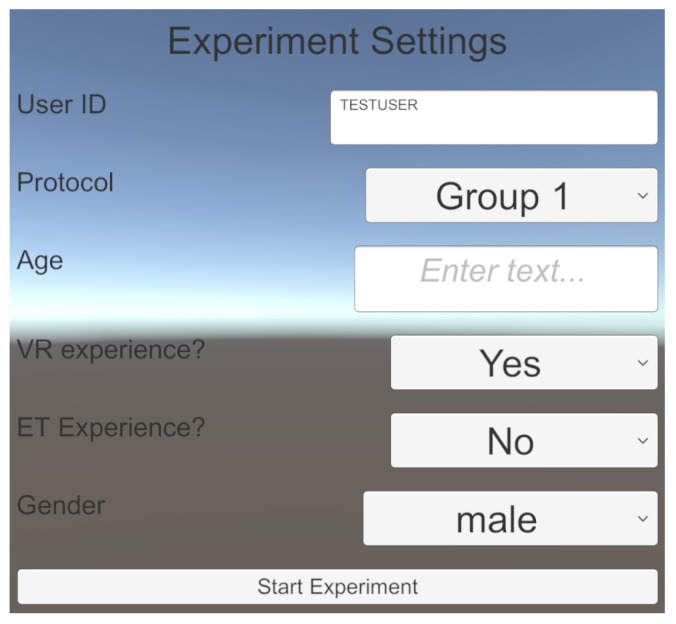
For every experiment, subject tracking is an essential part. The input mask is adaptable to incorporate the specific needs of the experimenter.

The organization of the experiment is essential for running experiments with multiple subjects. Therefore, VisionaryVR contains a sample scene MainMenu (Figure 6), which can be loaded at the beginning of each protocol. For each new run with a new subject, the experimenter can create a new subject folder named after the userID. In this example scene, the protocol for the current run can be picked, and basic demographic questions can be answered. The content is adjustable to the experiment’s needs. When starting the experiment, VisionaryVR creates a user folder inside the VisionaryVR global folder (which is changeable in the inspector). If the folder already exists, an incremented number is attached. This folder acts as the saving location for all user-related files.

#### 2.3.1. Dynamic Vision Task

**Figure 7:**
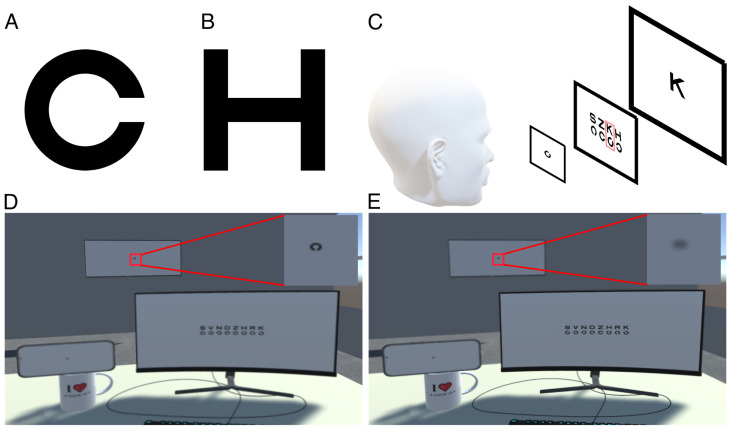
Components of the matching task with exemplary focus and defocus. (**A**) Landolt ring is the first type of stimulus used for the matching task. The opening gap can be oriented in eight different directions. (**B**) One of the eight used Sloan letters is presented on a second screen. (**C**) Stimuli are presented on three different screens at different distances. The closest screen represents a smartphone at a reading distance 30 cm. At an intermediate distance of 1 m, there is a screen representing a computer display. The visual performance for far vision is tested at 6 m distance. One of the screens shows a Landolt ring of random orientation, and a second screen shows a Sloan letter. A table of both stimuli types is displayed on the third screen. The task for the subjects is to answer if the two single stimuli are in the same table column on the third screen, corresponding to a match. This requires a focus shift between all three viewing distances. (**D**) Screenshot of the matching task in the virtual environment. Defocus blur is simulated considering the virtual focus controller’s local depth and dynamically set focus distance. In this example, the focus distance is set to the far screen. (**E**) The same scene with focus distance is set for the intermediate screen. The Landolt stimulus on the far screen is blurred and out of focus.

Visual performance is usually evaluated for a fixed distance only. This testing does not represent the challenges of natural vision with many dynamic changes in focus distance, which is especially difficult for presbyopes, even with optical correction. For dynamically evaluating performance, we designed a matching task with multiple viewing distances to simplify the VR scenarios into psychophysical paradigms that could be easily repeated. This task requires participants to shift their focus between screens displaying stimuli at various distances, simulating real-world situations involving dynamic gaze changes. Our simulator recreates a typical office environment with different viewing distances. Subjects are presented with stimuli on multiple virtual screens, requiring them to change their gaze dynamically. The VR simulation lets us dynamically set the virtual focus distance by considering depth information from the 3D scene, allowing for calculating realistic depth-dependent blur. By implementing a matching task that compares visual stimuli on different screens, we can objectively quantify visual performance without restricting the subjects’ natural gaze and head-movement behavior. This setup accurately represents real-world scenarios and allows for the precise measurement of visual performance. The stimuli on the screens are normally distributed and shown at the center or the corner of the screen to incorporate complex cases where a stimulus is close to the border of two distinct depths. The developed task with multiple viewing distances compares visual performance and convenience in a realistic everyday scenario with dynamic gaze changes. Stimuli were shown on three different screens at three different distances: a smartphone at 30 cm, a computer display at 1 m, and a far-distance TV screen at 6 m. This arrangement elicits many dynamic changes in viewing distance, as subjects must perform a matching task with stimuli placed on all three screens. Landolt rings, and Sloan letters are standardized optotypes for visual acuity testing to reduce task complexity. Subjects must compare if the given combination of Landolt ring and Sloan letter appeared in the same table column displayed on the third screen. Using a combination of two different types of stimuli, we reduce task complexity while maintaining sensitivity to defocus blur. Randomizing the screen displaying the table prevented subjects from following a fixed order of fixating the screens, resulting in dynamic and better comparable gaze changes to natural behavior. A simple visualization of the task in two different focus depths can be seen in Figure 7.

#### 2.3.2. Questionnaires

**Figure 8:**
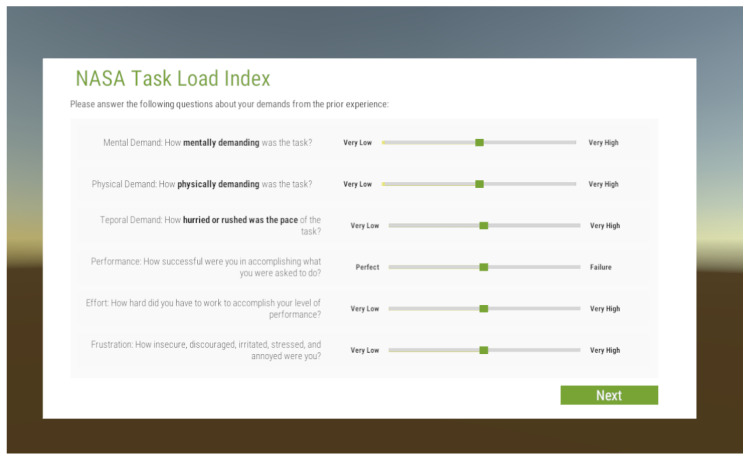
Provided as an abbreviation in the experiment controller (e.g., TLX), a questionnaire scene is loaded that is shown in VR for the subject. Depending on the abbreviation, the questions of the correct questionnaire are loaded dynamically into the canvas. Interaction takes place using VR controllers. The experimenter can see the same view on the screen during the whole scene.

To ensure the effectiveness and suitability of optical systems for everyday use, it is essential to replicate the natural behavior of individuals in real-world scenarios. Researchers can optimize the design and control mechanisms to meet users’ specific needs and preferences by studying how individuals interact with and adapt to optical methods. Psychophysical paradigms and behavioral quantifiers play a vital role in understanding visual perception and performance aspects, quantifying the benefits and limitations of new methods, and comparing their performance against traditional methods. Behavioral quantifiers also provide objective user experience and satisfaction measures, enabling researchers to gain valuable insights into users’ preferences and inform future improvements and customization of these devices. The VR questionnaire loader is implemented in a separate scene and loaded when mentioned in the protocol. Which questions to load can be decided by the choice of scene parameters in the experiment manager. The questionnaire to be used can be specified for each scene separately. As long as a .json file with the questions is stored, it can be loaded. The file is dynamically read out at runtime and displayed in virtual reality (see Figure 8 for an example visualization in VR). The user can operate the questions via ordinary VR controllers.

When a run is finished, the user folder contains a subfolder for each scene that has been shown. These are system-timestamped files written by single components, such as gaze files, autofocual controller tuning power changes, questionnaire answers, or task responses (reaction time, correct answers, and given answers). This organization leads to an optimal structure for offline analysis.

## 3. Discussion

This work introduces VisionaryVR, a groundbreaking VR-based simulation tool poised to transform vision science research. VisionaryVR empowers researchers to simulate a broad spectrum of visual impairments and optical corrections with unmatched realism and control, integrating seamlessly with eye-tracking devices, enabling the simulation of various refractive errors through dynamic focus adjustments, and incorporating a direct VR response questionnaire loader. The empirical validation of VisionaryVR has quantitatively demonstrated its efficacy in replicating a wide spectrum of visual impairments with high fidelity. This was achieved by developing a sophisticated rendering pipeline that dynamically adjusts simulations based on real-world optical properties. For example, our approach to simulating presbyopia involved integrating complex algorithms that adjust the depth of field in real-time based on the user’s focus point, thereby mimicking the natural deterioration of the close vision associated with age. Despite its innovative approach, VisionaryVR faces limitations, particularly the resolution of VR glasses, which have not yet achieved the fidelity necessary to mimic real-life scenarios perfectly. This limitation highlights a “last mile” in simulation accuracy that is currently unattainable, affecting the tool’s ability to replicate the full complexity of natural environments and subtly impacting the evaluation of visual impairments and correction solutions. VisionaryVR’s future iterations will seek to mitigate these limitations by enhancing its simulation capabilities to more accurately emulate conditions such as cataracts, glaucoma, and AMD. Efforts will address the vergence-accommodation conflict and the tool’s lack of mobility for outdoor environment simulation, enhancing configurability and exploring dynamic diopter changes. We anticipate technological advancements in VR hardware will gradually narrow the gap between simulated and real-world visual experiences. To address the challenge of outdoor simulation, we are incorporating environmental lighting models that replicate the variability in natural light conditions and their impact on visual perception. This includes simulating different times of day and weather conditions to study their effects on various visual impairments and correction methods. VisionaryVR distinguishes itself with its user-friendly design, adaptability, and comprehensive feature set, supported by its open-source nature and modular architecture. Beyond vision science, it holds potential benefits for fields like augmented reality and human-computer interaction, offering a valuable platform for validating depth estimation and eye-tracking algorithms.

## 4. Conclusions

VisionaryVR represents a significant leap in applying VR technology to vision science research, characterized by its innovative simulation capabilities. Offering sophisticated simulations of optical aberrations and corrections, bridging theoretical concepts with their practical applications, and deepening our understanding of vision impairments. It fosters enhanced experimental control by enabling the precise replication and manipulation of complex visual scenarios for conducting detailed, adaptable studies. Additionally, accelerating the development of innovative optical solutions and contributing to the broader vision science field. Despite challenges, like the vergence-accommodation conflict and resolution limitations, VisionaryVR’s achievements and potential underscore its indispensable role in advancing optical health and vision correction technologies. Its current and future contributions set a new standard for realism and applicability in vision science simulations, driving future research and practical applications. 

## Data Availability

The simulation tool’s source code and a unity package are publicly available at https://github.com/benedikt-hosp/visionaryVR (accessed on 1 April 2024).

## Abbreviations

The following abbreviations are used in this manuscript:

VR	Virtual Reality
ET	Eye-Tracking
TLX	Task-Load Index
